# Eplerenone for Central Serous Chorioretinopathy: Is There Still Room for This Treatment?

**DOI:** 10.3390/biomedicines14020368

**Published:** 2026-02-05

**Authors:** Irini Chatziralli, Chrysa Agapitou, Stamatios Lampsas, Alexandros Chatzirallis, Alexia Risi-Koziona, Rafaela Smarlamaki, Konstantinos Pappelis, George Theodossiadis, Panagiotis Theodossiadis

**Affiliations:** 2nd Department of Ophthalmology, Attikon University Hospital, National and Kapodistrian University of Athens, 12462 Athens, Greece; chr.agapitou@gmail.com (C.A.); lampsas.stam@gmail.com (S.L.); firmachat@gmail.com (A.C.); alexiarisi@gmail.com (A.R.-K.); rafaelasma@hotmail.com (R.S.); kpappelis91@gmail.com (K.P.); theodossiadisg@ath.forthnet.gr (G.T.); patheo@med.uoa.gr (P.T.)

**Keywords:** central serous retinopathy, eplerenone, treatment, subretinal fluid, visual acuity, retinal disease

## Abstract

**Background/Objectives**: The purpose of this study was to evaluate the efficacy and safety of oral eplerenone in patients with acute and chronic central serous chorioretinopathy (CSCR). **Methods**: In this prospective study, 43 patients with CSCR and subretinal fluid on optical coherence tomography (OCT) at baseline were divided either to oral eplerenone (n = 23) or observation (n = 20). All subjects underwent best-corrected visual acuity (BCVA) measurement, OCT, and fluorescein angiography (FA) at baseline. The changes in BCVA and subretinal resolution (SRF) were examined at 1, 6, and 12 months after the initiation of treatment. Potential adverse events were recorded. **Results**: At month 6, SRF resolution was observed in 78.3% and 45% of the patients in the eplerenone and control groups, respectively (*p* = 0.024). However, there was a recurrence of fluid in three patients in the eplerenone group and in four patients in the control group. Therefore, at month 12, 65.2% of the patients in the eplerenone group and 25% in the control group had SRF resolution (*p* = 0.008). There was a statistically significant improvement in BCVA at 6 months (*p* < 0.001) and 12 months (*p* < 0.001) in the eplerenone group, while in the control group, there was an improvement in BCVA at 6 months (*p* = 0.079) and 12 months (*p* = 0.259), which did not reach statistical significance. Regarding adverse events, no ocular nor systemic adverse events were reported during the follow-up period, apart from dry mouth in 7 out of 23 patients (30.4%) taking eplerenone. **Conclusions**: Oral eplerenone was found to be a safe and effective treatment alternative for the management of CSCR in both acute and chronic cases, providing SRF resolution in approximately 65% of patients with significant improvement in visual acuity at the 12^th^ month of follow-up.

## 1. Introduction

Central serous chorioretinopathy (CSCR) is a disorder affecting the choroid and retina, marked by localized serous elevation of the neurosensory retina and/or the retinal pigment epithelium (RPE), leading to the buildup of subretinal fluid, primarily affecting the macular region [1,2]. The etiology of CSCR is thought to be due to congestion of choroidal vessels, leading to a pachychoroid phenotype within the spectrum of venous overload choroidopathy [1,2,3]. It typically affects young to middle-aged men and is associated with a variety of systemic and behavioral risk factors, such as psychological stress, type A personality, corticosteroid use, hypertension, obstructive sleep apnea, *Helicobacter pylori* infection, smoking, alcohol consumption, and oxidative stress [2,4,5]. Patients with CSCR may be asymptomatic; however, if the disease affects the central macula, symptoms may include central vision blurring, metamorphopsia, micropsia, and a central scotoma. There are two forms of CSCR: acute and chronic, which differ in terms of symptom duration and RPE integrity. The former typically presents as subretinal fluid (SRF) and commonly pigment epithelium detachment (PED) without RPE defects, whereas the latter is associated with RPE defects and variable photoreceptor degeneration [6]. Particularly, the development of acute CSCR is primarily driven by choroidal vascular abnormalities that involve choroidal hyperpermeability and venous congestion resulting in elevated hydrostatic pressure, overwhelming of the RPE [7]. This pressure disrupts the RPE’s barrier and pump function, causing focal leaks and accumulation of subretinal fluid beneath the macula which reflects in a RPE pressure-induced reversal of its pump activity [3,8]. Chronic CSC is distinguished by persistent choroidal vascular hyperpermeability that results in chronic dysfunction and degeneration of the RPE causing persistent neurosensory retinal detachment and subretinal fluid [8,9]. These changes in chronic CSC are associated with key choroidal alterations that include increased choroidal thickness, enlarged choroidal vessels, and intervortex venous anastomoses, all of which promote venous congestion and reduced outflow [10].

Multiple therapeutic approaches have been suggested for CSCR, among which verteporfin photodynamic therapy (PDT) appears to be the most effective, particularly in cases of chronic disease [2,3]. Nevertheless, numerous alternative treatment approaches have also been proposed, including laser photocoagulation, mineralocorticoid receptor antagonists, intravitreal anti-vascular endothelial growth factor (anti-VEGF) agents, carbonic anhydrase inhibitors, beta-blockers and antioxidants [2,3,11]. The acute form of CSCR is usually self-limiting, characterized by spontaneous resolution of retinal detachment and restoration of visual function within three months. However, chronic cases are commonly refractory to treatment, and may result in permanent and significant visual impairment [1,2,3,11].

Eplerenone has been investigated in chronic CSCR due to its selective inhibition of the mineralocorticoid receptor, whose activation is thought to contribute to choroidal vascular hyperpermeability and CSCR pathogenesis [12]. Several studies have explored the efficacy of eplerenone in CSCR management with controversial results [3]. Results from the multicenter randomized VICI trial showed that eplerenone was not associated with superior visual acuity or subretinal fluid resolution compared to placebo after 12 months [13]. In addition, findings from the SPECTRA trial showed that half-dose photodynamic therapy outperformed oral eplerenone in both effectiveness and safety for chronic CSCR [14,15]. Nonetheless, real-world evidence has suggested favorable outcomes with oral eplerenone in the treatment of CSCR [6,16,17,18,19].

Based on the foregoing, the objective of the present study was to evaluate the efficacy and safety of oral eplerenone in patients with both acute and chronic CSCR.

## 2. Materials and Methods

This prospective study included 43 treatment-naïve patients with acute (duration < 3 months) or chronic CSCR and evidence of subretinal fluid on optical coherence tomography (OCT), who were evaluated and treated at the Second Department of Ophthalmology, National and Kapodistrian University of Athens, Greece. All participants were adults aged 18 years or older. Exclusion criteria were retinal diseases other than CSCR, intraocular inflammation, any previous treatment including PDT, anti-VEGF injections, laser photocoagulation, any contraindication for eplerenone use such as severe renal and heart failure, pregnancy, concomitant administration of potassium-sparing diuretics, angiotensin-converting enzyme inhibitors (ACE inhibitors), or angiotensin receptor blockers (ARBs). These patients were excluded to avoid potential confounding effects on serum potassium levels and renal function, both of which were key safety considerations in this study. Moreover, these patients were excluded to ensure that any observed safety or efficacy outcomes could be attributed specifically to eplerenone without interference from concomitant renin–angiotensin system blockade. In addition, patients with choroidal neovascularization were excluded based on the fundus fluorescein angiography (FFA) findings. The study was performed in compliance with the principles of the Declaration of Helsinki and received approval from the Institutional Review Board of Attikon University Hospital.

At baseline, all patients underwent assessment of best-corrected visual acuity (BCVA) using Snellen charts, along with dilated fundus examination, spectral-domain optical coherence tomography (SD-OCT), and fluorescein fundus angiography (FFA) performed with the Heidelberg Spectralis system (HRA+OCT, Heidelberg, Germany). Participants were alternatively divided into two groups: Group I (n = 23), in which patients received oral eplerenone (Inspra, Pfizer, New York, NY, USA) at a dose of 25 mg/day for 2 weeks and 50 mg/day thereafter for a total of 6-month treatment, if potassium levels were within normal limits at the beginning of week 3, and Group II (n = 20), in which patients did not receive any treatment (control group). The off-label use and potential risks and benefits of eplerenone were explained to all subjects in Group I before entering the study. All assessments and measurements were performed by experienced clinicians who were formally masked to treatment allocation.

Patients were followed up with at 1, 3, 6, and 12 months after baseline with BCVA measurement and SD-OCT, while FFA was performed at the physician’s discretion. Primary outcomes were the percentage of patients achieving SRF resolution on SD-OCT and the change in BCVA at 6 and 12 months of follow-up for both groups. In SRF resolution, the complete subretinal fluid resolution was evaluated, which means the subretinal fluid has fully disappeared on SD-OCT. A baseline metabolic panel was obtained to confirm normal potassium levels, and therapy was withheld if serum potassium was >5.5 mEq/L. Potential adverse events were recorded. All subjects provided informed consent before participating in the study. All potential adverse events, treatment options and patients’ questions were fully explained and answered.

### Statistical Analysis

All statistical analyses were carried out with SPSS version 28.0 (IBM Statistics, Armonk, NY, USA). Descriptive data were reported as mean values with corresponding standard deviations (SD). Continuous variables were compared using the Mann–Whitney–Wilcoxon test, while categorical variables were analyzed with the chi-square test. Comparisons across different time points were carried out using the Wilcoxon signed-rank test. As three pairwise comparisons were performed, Bonferroni correction was applied to account for multiple testing. Statistical significance was defined as *p* < 0.05, except in analyses adjusted using the Bonferroni method.

## 3. Results

Baseline demographic and clinical characteristics of the study participants are presented in Table 1. The mean age of subjects was 51.8 ± 8.3 years. A total of 40 out of 43 patients were male (93.0%) and 3 (7.0%) females. Of note, 37.2% presented with acute CSCR and 62.8% with chronic. All patients had SRF at baseline and 14 out of 43 patients (32.6%) also had pigment epithelium detachment (PED). A total of 20.9% of patients presented ellipsoid zone disruption on SD-OCT. On FFA, 35 out of 43 patients (81.4%) had hyperreflective areas, consistent with active leakage points due to CSCR. Patients with CNV were not included in this analysis. The mean BCVA was 0.41 ± 0.10 (decimal scale) and the mean central subfoveal thickness (CST) was 349.2 ± 49.3 μm. The two groups did not differ in between at baseline.

At month 6, there was SRF resolution in 18 out of 23 patients (78.3%) in the eplerenone group and in 9 out of 20 patients (45%) in the control group. However, there was a recurrence of fluid in three patients in the eplerenone group and in four patients in the control group. At month 12, 15 out of 23 patients (65.2%) in the eplerenone group and 5 out of 20 patients (25%) in the control group had SRF resolution. The two groups differed significantly regarding SRF resolution at month 6 (*p* = 0.024) and month 12 (*p* = 0.008).

**Figure 1:**
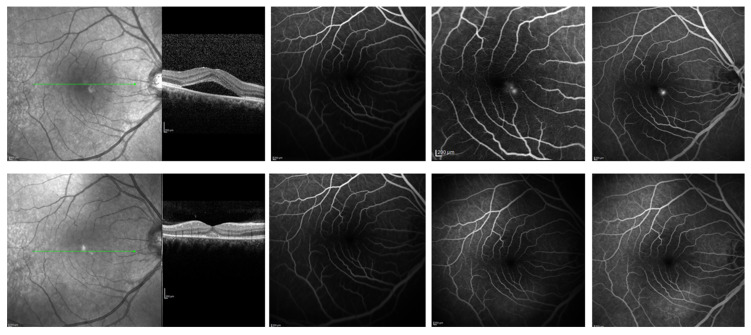
A 38-year-old male patient, who presented with chronic central serous chorioretinopathy in the right eye with subretinal fluid confirmed on optical coherence tomography and leakage point on fluorescein angiography at baseline (upper panel); optical coherence tomography of the same patient at month 12 after oral eplerenone, showing resolution of fluid, while fluorescein angiography has no leaking areas (bottom panel).

**Figure 2:**
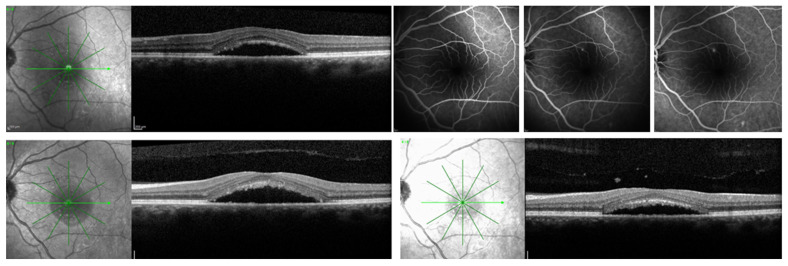
A 35-year-old male patient, who presented with chronic central serous chorioretinopathy in the left eye with subretinal fluid confirmed on optical coherence tomography and leakage point on fluorescein angiography at baseline (upper panel); optical coherence tomography of the same patient, who was observed at month 6 (bottom panel, left) and at month 12 (bottom panel, right), showing presence of fluid.

As far as BCVA is concerned, there was a statistically significant improvement in BCVA at month 6 (0.63 ± 0.10, *p* < 0.001) and at month 12 (0.59 ± 0.11, *p* < 0.001) in the eplerenone group. In control group, there was an improvement in BCVA at month 6 (0.45 ± 0.11, *p* = 0.079) and at month 12 (0.43 ± 0.12, *p* = 0.259), which did not reach statistical significance. The two groups differed significantly in terms of BCVA change at month 6 (*p* < 0.001) and at month 12 (*p* < 0.001) in favor of eplerenone. Figure 1 and Figure 2 show two representative cases of our study.

Regarding adverse events, neither ocular nor systemic adverse events were reported during the follow-up period, apart from dry mouth in 7 out of 23 patients (30.4%) taking eplerenone. All patients maintained serum potassium levels within normal range and renal function throughout the study.

## 4. Discussion

This study primarily demonstrated that oral eplerenone may benefit patients with CSCR, with around 65% achieving SRF resolution and significant BCVA improvement at 12 months, outperforming non-treatment, which resulted in only 25% resolution. Accordingly, patients treated with eplerenone presented a significant improvement in BCVA at month 12 compared with those who were not treated.

The primary goal of therapy for acute CSCR is to decrease choroidal vascular permeability and/or seal the RPE leakage. Based on this assumption, focal laser therapy and PDT with verteporfin are considered the mainstay of treatment for acute CSCR [19]. However, focal laser treatment is not recommended when the leak is located too close to the fovea [3]. Photodynamic therapy (PDT), whether full- or reduced-fluence, has been shown to effectively decrease choroidal vascular permeability and SRF [3]. It is worth mentioning that PDT can be associated with adverse events such as RPE atrophy, development of CNV, and choroidal ischemia [17,20]. In addition, since July 2021, there has been a worldwide shortage of verteporfin (Visudyne^®^, QLT Inc., Vancouver, BC, Canada), an essential medicine required for PDT [21]. A questionnaire distributed among retinal specialists indicated that the shortage of verteporfin had a major impact on ophthalmic care worldwide and was regarded as a serious problem [21]. Due to the limited availability of verteporfin, exploring alternative therapies is crucial in the management of CSCR. Oral eplerenone, which targets the choroidal mineralocorticoid receptor pathway and decreases choroidal vascular permeability, represents a potential option, although its efficacy remains debated [3,6].

As mentioned above, the VICI trial showed no superiority of eplerenone over observation in patients with CSCR at the one-year follow-up [13]. Accordingly, the authors concluded that oral eplerenone should be avoided in the treatment of CSCR cases [13]. However, there were several methodological concerns related to the study design, patients’ inclusion and exclusion criteria, and duration of treatment with eplerenone. Specifically, cases with CNV do not seem to be excluded, but the presence of CNV complicating CSCR is a major negative predictor of response to eplerenone [22]. Additionally, in the treatment group, eplerenone was stopped when SRF resolution occurred; thus, 30% of patients received less than 3 months eplerenone [13,23]. In other studies showing good results with eplerenone in CSCR, the treatment was continued for 6–12 months, while CSCR recurred when treatment ceased [12,23,24]. As a result, Boscia et al. suggested continuous eplerenone treatment for chronic CSCR, showing beneficial effects in SRF resolution even after four years [17]. However, other studies recommend that eplerenone was not superior to placebo in improving visual acuity or anatomical outcomes in chronic CSCR after 12 months of treatment [13]. A recent meta-analysis shows that mineralocorticoid receptor antagonists can yield short-term anatomical benefits leading to reduction in mean SRF height and improvement in SRF resolution at 1 month, but their long-term effectiveness is limited [18]. Although eplerenone has been suggested to improve outcomes in CSC, the results across studies remain inconsistent, prompting us to investigate its effects further in this study.

The results of our study, including both cases of acute and chronic CSCR, are in line with those of previous real-world studies. In a prospective case–control study by Venkatesh et al., including 58 patients with unilateral acute CSCR, complete SRF resolution was noted in 62% of patients treated with eplerenone 3 months after the initiation of treatment, while 31% of patients in the control group had SRF resolution at month 3. In addition, eplerenone was found to achieve faster vision improvement compared to observations [19]. In another study by Zucchiati et al., 80% of eyes with acute CSCR treated with eplerenone showed complete SRF resolution at 3 months [25].

In the context of chronic CSCR, Bousquet et al. found that 75% of patients treated with eplerenone achieved complete resolution of subretinal fluid (SRF) at 3 months of follow-up [26]. These findings align with those of Singh et al. and Salz et al., who observed complete SRF resolution in 35% and 64% of patients, respectively, after six months of eplerenone therapy [27,28]. Other prospective studies have reported comparable findings, although their follow-up was limited to six months or less [29,30,31]. A meta-analysis by Wang et al. concluded that mineralocorticoid antagonists were effective and improved visual acuity in patients [32]. In contrast, Petkovsek et al. examined the efficacy of eplerenone in 83 patients with chronic CSCR and found that only a minority of patients had complete resolution of SRF, but the primary beneficial effect of eplerenone occurred within the first year of treatment [12].

Notably, predictive factors may influence the response to eplerenone treatment. Bousquet et al. showed that a thicker baseline choroid (>515 mm) was associated with a more favorable treatment response [26], whereas Fraenkel et al. pointed out that stress is a predictive factor [33]. Moreover, Sahu et al. found that the absence of PED and double-layer sign on OCT were associated with a good response [16]. Borelli et al. reported similar findings, identifying thicker subfoveal choroidal thickness, smaller maximum SRF diameter, fewer serous PEDs, and a lower number of intraretinal hyperreflective foci as independent predictors of complete SRF resolution at three months [34].

Eplerenone is associated with a risk of hyperkalemia, particularly in patients with renal insufficiency, advanced heart failure, advanced age, or when used in combination with other medications that affect potassium balance [35]. Nonetheless, adverse effects are generally related to the dosage and are reversible after stopping treatment [35]. In our study, approximately 30% of the patients exhibited dry mouth, which was not a significant adverse event.

A study limitation is the absence of an in-depth assessment of anatomical and clinical variables that could influence or predict response to treatment. Furthermore, it would be of interest to assess contrast sensitivity or microperimetry, to better investigate the impact of eplerenone on functional results, since only basic outcome measures were conducted. Moreover, we did not differentiate between unilateral and bilateral disease, which may represent a limitation when interpreting the generalized treatment response, as bilateral CSC may reflect a more severe or chronic phenotype with potentially different pathophysiological mechanisms, prognosis, and responsiveness to mineralocorticoid receptor antagonism. A further limitation is the absence of enhanced depth imaging OCT (EDI-OCT), as the use of a standard SD-OCT protocol may have limited detailed assessment of choroidal thickness and choroidal structural changes relevant to disease characterization and treatment response. Also, due to the relatively small sample size and the absence of predefined power calculation, subgroup analyses comparing acute and chronic CSCR were not performed, which could restrict the applicability of our findings to these specific subtypes. However, this prospective study with a 12-month follow-up period enabled the assessment of both short-term and longer-term outcomes, which is important given the variable course of CSCR and efficacy and safety in a real-world clinical context of eplerenone.

## 5. Conclusions

Based on our findings, oral eplerenone may provide a safe and effective treatment alternative for the management of CSCR in both acute and chronic cases, with SRF resolution observed in approximately 65% of patients at the 12^th^ month of follow-up compared to 25% in the observation group at the 12^th^ month of follow-up and significant improvement in visual acuity. However, these findings must be interpreted with caution and large-scale studies are needed to determine which patients are suitable for eplerenone treatment and may respond well to this treatment.

## Figures and Tables

**Table 1:** Baseline characteristics, both demographic and clinical, of the study sample at baseline.

	Patients (n = 43)	Group I (n = 23)	Group II (n = 20)	*p* Value
Age (mean ± SD, years)	51.8 ± 8.3	47.5 ± 9.1	52.3 ± 7.9	0.074
Gender (n, %)				
Male	40 (93.0%)	21 (91.3%)	19 (95.0%)	0.635
Female	3 (7.0%)	2 (8.7%)	1 (5.0%)	
Duration				
Acute (≤3 months)	16 (37.2%)	6 (26.1%)	11 (55.0%)	0.053
Chronic (>3 months)	27 (62.8%)	17 (73.9%)	9 (45.0%)	
Pigment Epithelium Detachment (n, %)	14 (32.6%)	8 (34.8%)	6 (30.0%)	0.739
Ellipsoid zone disruption (n, %)	9 (20.9%)	5 (21.7%)	4 (20.0%)	0.889
Best-corrected visual acuity (mean ± SD, decimal)	0.41 ± 0.10	0.42 ± 0.11	0.39 ± 0.10	0.358
Central subfoveal thickness (mean ± SD, μm)	349.2 ± 49.3	339.5 ± 47.2	352.1 ± 51.9	0.410

## Data Availability

The original contributions presented in this study are included in the article. Further inquiries can be directed to the corresponding author.
